# The Effects of Mechanical Stretch on Integrins and Filopodial-Associated Proteins in Normal and Glaucomatous Trabecular Meshwork Cells

**DOI:** 10.3389/fcell.2022.886706

**Published:** 2022-04-29

**Authors:** Yong-Feng Yang, Ying Ying Sun, Donna M. Peters, Kate E. Keller

**Affiliations:** ^1^ Casey Eye Institute, Oregon Health & Science University, Portland, OR, United States; ^2^ Department of Pathology and Laboratory Medicine, University of Wisconsin School of Medicine and Public Health, Madison, WI, United States; ^3^ Department of Chemical Physiology and Biochemistry, Oregon Health & Science University, Portland, OR, United States

**Keywords:** glaucoma, trabecular meshwork, filopodia, integrins, tunneling nanotubes, myosin-X, cadherin-11, mechanosensing

## Abstract

The trabecular meshwork (TM) is the tissue responsible for regulating aqueous humor fluid egress from the anterior eye. If drainage is impaired, intraocular pressure (IOP) becomes elevated, which is a primary risk factor for primary open angle glaucoma. TM cells sense elevated IOP via changes in their biomechanical environment. Filopodia cellular protrusions and integrin transmembrane proteins may play roles in detecting IOP elevation, yet this has not been studied in detail in the TM. Here, we investigate integrins and filopodial proteins, such as myosin-X (Myo10), in response to mechanical stretch, an *in vitro* technique that produces mechanical alterations mimicking elevated IOP. Pull-down assays showed Myo10 binding to α5 but not the β1 subunit, αvβ3, and αvβ5 integrins. Several of these integrins colocalized in nascent adhesions in the filopodial tip and shaft. Using conformation-specific antibodies, we found that β1 integrin, but not α5 or αvβ3 integrins, were activated following 1-h mechanical stretch. Cadherin -11 (CDH11), a cell adhesion molecule, did not bind to Myo10, but was associated with filopodia. Interestingly, CDH11 was downregulated on the TM cell surface following 1-h mechanical stretch. In glaucoma cells, CDH11 protein levels were increased. Finally, mechanical stretch caused a small, yet significant increase in Myo10 protein levels in glaucoma cells, but did not affect cellular communication of fluorescent vesicles via filopodia-like tunneling nanotubes. Together, these data suggest that TM cell adhesion proteins, β1 integrin and CDH11, have relatively rapid responses to mechanical stretch, which suggests a central role in sensing changes in IOP elevation *in situ*.

## Introduction

Glaucoma is a leading cause of blindness, with a global prevalence of 3.54% in the population aged 40–80 years (Tham et al., 2014). A major risk factor of glaucoma is elevated intraocular pressure (IOP), which is caused by dysfunction in the trabecular meshwork (TM) aqueous humor drainage pathway (Stamer and Acott, 2012). The extracellular matrix (ECM) of TM tissue is a source of outflow resistance and glaucomatous ECM differs in amount, structure and organization compared to normal TM tissue (Fuchshofer and Tamm, 2009; Tektas and Lutjen-Drecoll, 2009; Vranka et al., 2015; Acott et al., 2021; Keller and Peters, 2022). Glaucomatous TM tissue has altered biomechanics compared to age-matched tissue derived from non-glaucomatous TM tissue (Last et al., 2011; Raghunathan et al., 2018) Notably, glaucoma TM cells in culture display molecular memory and synthesize and assemble dysfunctional matrices similar to those found *in situ* (Raghunathan et al., 2018; Acott et al., 2021; Wirtz et al., 2021). During a normal homeostatic response to elevated IOP, stretch and/or distortion of transmembrane mechanosensors activate downstream signaling pathways, which ultimately adjust aqueous outflow (Acott et al., 2021). Integrins have been implicated in the detection of elevated IOP (Gagen et al., 2014; Filla et al., 2017; Faralli et al., 2019). Their extracellular domains bind ECM molecules such as fibronectin and collagens, thus sensing alterations in extracellular biomechanical cues caused by elevated IOP. Integrins then switch from a bent, inactive conformation to an active state (Filla et al., 2017). Mechanical forces activate α5β1, or αvβ3 integrins, in fibroblasts and endothelial cells, respectively (Tzima et al., 2001; Katsumi et al., 2005). Yet, this has not been studied in TM cells. A tightly coordinated regime of cellular mechanosensing, mechanotransduction, and ECM remodeling is required to precisely coordinate homeostatic responses to elevated IOP.

Filopodia are projections that extend from the cell surface and are important for mechanosensing, phagocytosis, and fundamental cellular processes such as cell adhesion, migration, spreading, division and growth factor signaling (Mattila and Lappalainen, 2008). Using live cell imaging, we previously showed that highly dynamic filopodia emanate from TM cells and these can extend over long distances (> 100 µm) in cells and tissue (Keller et al., 2017; Sun et al., 2019a; Keller and Kopczynski, 2020). Filopodia growth involves multiple steps and various proteins are found at the base, the shaft, or at the tip of the filopodia (Mattila and Lappalainen, 2008). The identity and location of filopodial proteins is cell-type dependent (Ljubojevic et al., 2021). Filopodial base proteins include Arp2/3, filopodial shaft proteins include β1 integrins, epidermal growth factor receptor pathway 8 (Eps8), and fascin, while proteins found at filopodial tips include myosin-X (Myo10), mDia2 (also known as Diaph3), and β1-integrin (Mattila and Lappalainen, 2008; Watanabe et al., 2010). Interestingly, in U2OS cells, Myo10 binds to α5β1 integrin, where it functions to transport these integrin subunits toward to the filopodial tip (Miihkinen et al., 2021). Several cadherins are also involved in filopodia formation and extension in endothelial and neuronal cells, as well as in mouse embryos (Almagro et al., 2010; Fierro-Gonzalez et al., 2013; Lai et al., 2015). Cadherin-11 (CDH11), also known as OB-cadherin, plays a role in cell-cell adhesion and cell-ECM adhesion (Langhe et al., 2016), and it has strong expression in TM cells where it is up-regulated by TGFβ2 (Wecker et al., 2013; Webber et al., 2018). However, CDH11 has not been investigated regarding filopodia in TM cells.

Tunneling nanotubes (TNTs) are filopodia-like structures that are important for cellular communication (Ljubojevic et al., 2021). Since their discovery in 2004, TNTs have been described in many cells types where they transport large cellular organelles (lysosomes, mitochondria, endosomes) and smaller cargoes (viruses, microRNAs, signaling molecules) between neighboring cells through a tube connecting their cytoplasms (Rustom et al., 2004). We have described TNTs in normal TM cells as well as glaucoma TM cells (Keller et al., 2017; Sun et al., 2019a). Communication via TNTs is advantageous in the aqueous environment of the anterior eye since cargoes can be directly transported between cells and signals are not diluted in aqueous fluid. Moreover, TNTs can extend up to 100 µm in tissue, which allows cells to communicate over long distances (Keller et al., 2017; Ljubojevic et al., 2021). This potentially allows cells located on separate TM beams to coordinated responses to elevated IOP *in situ*. Myo10 is a quintessential filopodial protein that is important for TNT formation (Berg and Cheney, 2002; Bohil et al., 2006; Watanabe et al., 2010; Gousset et al., 2013). Myo10 protein distribution is altered in glaucomatous TM tissue and Myo10 gene silencing reduces outflow through the TM (Sun et al., 2019a; Sun et al., 2019b). This implicates a role for Myo10 in IOP homeostasis.

Filopodia and integrins are mechanosensors likely involved in sensing changes to the TM biomechanical environment caused by elevated IOP. Therefore, we investigated the effects of mechanical stretch on filopodia-associated proteins, Myo10 protein interactions, and cellular communication via TNTs in normal and glaucomatous TM cells.

## Methods

### Primary Trabecular Meshwork Cell Culture

Normal (NTM) and glaucoma (GTM) cells were cultured from TM tissue dissected from human cadaver eyes (Lions VisionGift, Portland, OR) using established techniques (Keller et al., 2018). Donor information was de-identified prior to use and use of cadaver eyes was considered exempt by OHSU Institutional Review Board. Demographics of human donor eyes are in Supplementary Table S1. All TM cells were used prior to passage six and were characterized by monitoring cell phenotype by phase microscopy, and by induction of myocilin protein by dexamethasone treatment (Supplementary Figure S1) (Keller et al., 2018). Characterization for some cell strains are already published (Sun et al., 2019a; Sun et al., 2019b).

### Co-Immunoprecipitation Assays

Myosin-10 polyclonal antibody was incubated overnight with protein A/G Magnetic Beads (Pierce). Following washing with phosphate buffered saline with 0.05% Tween (PBST) and blocking with bovine serum albumin, the Myo10-antibody beads were incubated with TM RIPA lysates with 10 mM MgCl_2_ overnight at 4°C. The tubes were then placed in a magnetic holder so Myo10 antibody-coated magnetic beads remained in the tube while they were washed thoroughly with PBST. After the final wash, 1x SDS-PAGE sample buffer was added to the tube, samples were boiled, and immunoprecipitated proteins were separated by SDS-PAGE. Following transfer, nitrocellulose membranes were probed with either integrin β1, α5, αvβ3, αvβ5, or CDH11 primary antibodies (Supplementary Table S2). Secondary antibodies were IRDye 700-conjugated anti-rabbit or IRDye 800-conjugated anti-mouse antibodies (Rockland). Membranes were scanned using an Odyssey gel imaging system (Licor, Lincoln, NE, United States).

### Mechanical Stretch

Mechanical stretch experiments were performed as described previously (Bradley et al., 2001). Briefly, TM cells were seeded on 6-well collagen type I-coated silicone membranes (FlexCell International, Burlington, NC) and grown to confluence. Cells were made serum-free and the membrane was stretched over a pushpin head for either 1 h (immunohistochemistry experiments) or 24 h (Western immunoblots). The upward inversion of the membrane with the cells attached produces a static radial and circumferential stretch of approximately 10% (Bradley et al., 2001). For comparison, an IOP of 30 mmHg was estimated to produce a mechanical stretch of outflow pathway cells of up to 50% *in situ* (Grierson and Lee, 1977).

### Immunohistochemistry and Confocal Microscopy

To investigate filopodial proteins, immunohistochemistry was performed. One hour prior to fixation, SiR-actin (Cytoskeleton, Inc.) and 10 µM verapamil, which increases fluorescent signal of SiR-actin, was added to the cultures (Keller and Kopczynski, 2020). Silicone membranes were removed from the plates and fixed with 4% paraformaldehyde. Following permeabilization in 0.03% Tween-20 and blocking with CAS-block (Invitrogen), primary antibodies (Supplementary Table S2) were incubated. After washing, Alex Fluor 488- or 594- conjugated species-specific secondary antibodies were incubated and coverslips were mounted on top of silicone membranes in DAPI-containing ProLong gold (Invitrogen). Fluorescence images were captured using an Olympus FV1000 confocal microscope using identical acquisition settings for each treatment (e.g., stretch vs. non-stretch). For CDH11 immunostaining, TM cells were graded as having high CDH11, medium, or low/no CDH11 based on fluorescence intensity. Grading was performed independently by two authors in a masked manner. To quantitatively assess integrin activation, Image J was used to measure total fluorescence in a field and the number of DAPI-stained nuclei was counted. Total activated integrin fluorescence was normalized for the number of nuclei, then data were averaged and a standard error calculated.

### Western Immunoblotting

RIPA lysates were harvested on ice from confluent NTM and GTM cells, or from TM cells that were mechanically stretched for 24 h. A 24 h time point was used so that changes in protein levels could accumulate. After harvesting, protease cocktail inhibitor was added to the RIPA lysates, proteins were separated by SDS-PAGE and Western immunoblots were performed with the indicated primary antibodies (Supplementary Table S2). Total ERK1 or tubulin antibodies were used as a loading control. Secondary antibodies were IRDye 700-conjugated anti-rabbit or IRDye 800-conjugated anti-mouse antibodies. Membranes were scanned using an Odyssey gel imaging system. Bands were quantitated from each lane of the immunoblots using ImageJ software and relative fluorescent units (RFUs) data were normalized for loading. For comparing mechanical stretch, RFUs of mechanically stretched TM cells were made a percentage of non-stretched RFUs for each cell strain. Data were averaged and plotted on the graph.

### Tunneling Nanotubes Vesicle Transfer Assay

A vesicle transfer assay quantitated fluorescent vesicle transfer via TNTs between cells (Abounit et al., 2015; Keller et al., 2017; Sun et al., 2019a) Briefly, cells were labeled with 1 µM of either Vybrant DiO (488 nm) or DiD (647 nm) dyes (ThermoFisher), mixed 1:1, and plated at 1 × 10^5^ cells/ml on FlexCell membranes. Cells were allowed to adhere for 2 h and then the membranes with attached cells were mechanically stretched for 24 h. Cells were fixed and immunostained with rat monoclonal anti-CD44, clone IM-7 (Stem Cell Technologies, Vancouver, BC) and Alexa-fluor 594-conjugated donkey anti-rat secondary antibody (ThermoFisher) to visualize the cell membrane. Confocal z-stacked images were acquired and the total number of DiO (green) and DiD (red)-labeled cells in each image were counted. The number of TM cells containing at least five vesicles of the opposite color (transferred vesicles) was then made a percentage of total cell number, as described previously (Keller et al., 2017; Sun et al., 2019a).

### Statistics

Biological (>*n* = 3) and technical (>*n* = 3) replicates were performed for all the experiments. The n’s are reported in the figure legends. Data are reported as mean ± standard error. One-way ANOVA with a post-hoc Tukey test, or a paired Students t-test, were performed. Significance was set at *p* < 0.05.

## Results

### Integrins and Myosin-X Interactions

**FIGURE 1:**
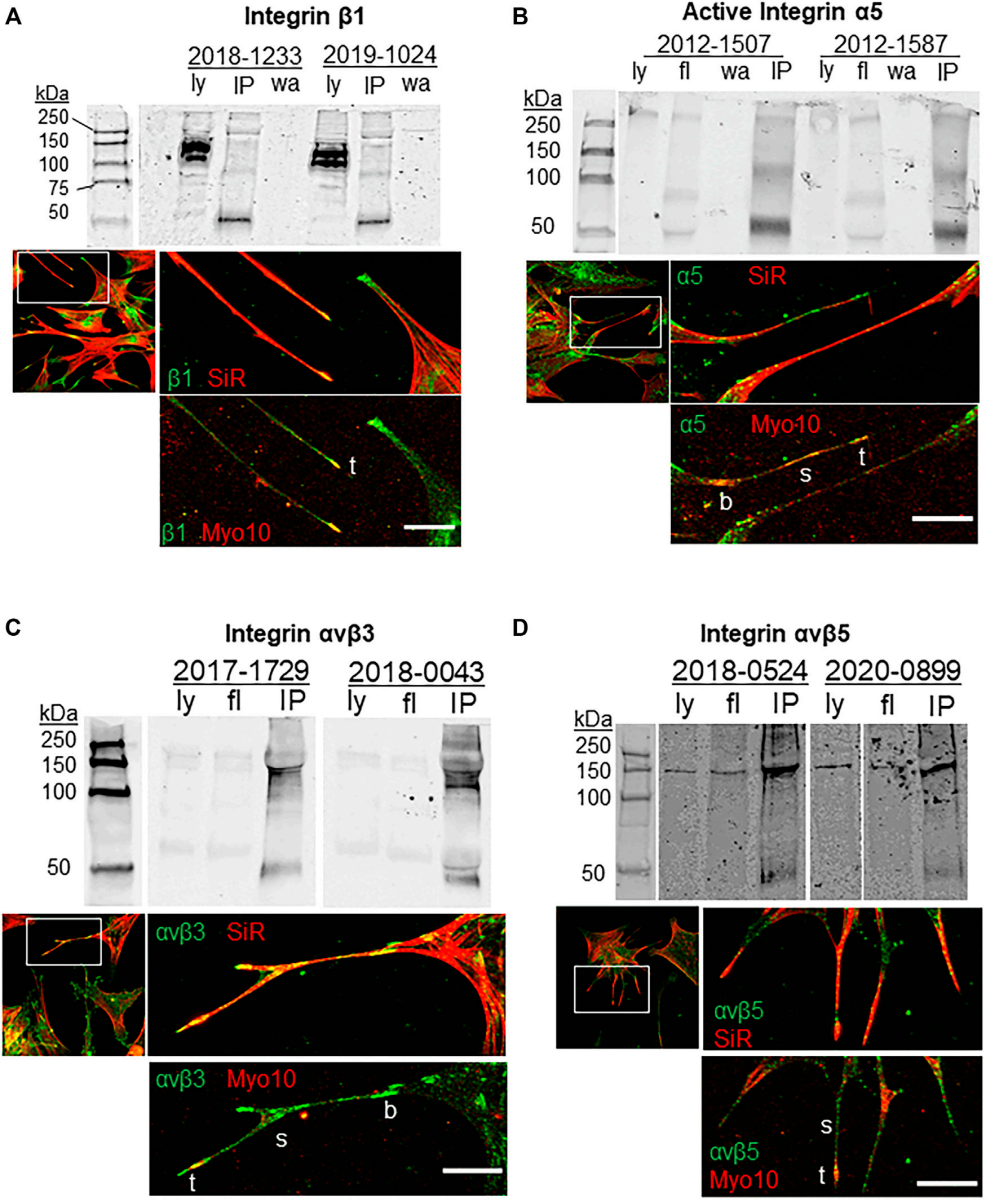
Integrins, Myo10 and filopodia. Co-immunoprecipitation assays investigated the binding of Myo10 to **(A)** β1-integrin **(B)** α5-integrin, **(C)** αvβ3 integrin, and **(D)** αvβ5 integrin. Myo10 was used to pull-down interacting proteins and Western immunoblots were probed with integrin antibodies. Pull-downs of two biological replicates are shown, which are representative immunoblots from at least four cell strains used for each integrin. Ly = lysate; fl = flow through; IP = co-immunoprecipitation; wa = wash. For immunofluorescence, TM cells were labeled with SiR-actin, then fixed, immunostained with integrin and Myo10 antibodies and confocal images were acquired. b = base; s = shaft; t = tip. Scale bar = 20 µm.

Previous studies indicated that β1 integrins and α5 integrins are transported along filopodia by Myo10 (Zhang et al., 2004; He et al., 2017; Miihkinen et al., 2021). Here, we investigated the co-localization of integrins with Myo10 in filopodia of TM cells and performed co-immunoprecipitation assays to examine Myo10-integrin protein interactions (Figure 1). β1 integrin strongly colocalized with Myo10 at the tips of filopodia, while along the shaft the β1 fluorescent signal was lower and there were only a few areas of colocalization (Figure 1A). Activated α5 integrin showed colocalization with Myo10 along the shaft and at the filopodia tip (Figure 1B). The localization of αvβ3 and αvβ5 integrins were also examined. There was a strong fluorescence signal for αvβ3 integrin along the filopodial shaft and at the tip (Figure 1C), but the signal for αvβ5 integrin showed much lower fluorescence (Figure 1D) suggesting that there were lower levels of αvβ5 integrin in filopodia.

To determine if Myo10 and these integrins interacted, we performed pull-down assays using a Myo10 antibody. As shown in Figure 1, α5 (116 kDa), β3 and β5 integrin subunits (∼135 kDa) were pulled down using the Myo10 antibody, but we failed to show an interaction between Myo10 and the β1 integrin subunit (140 kDa). All pull-down lanes showed a band at ∼50 kDa, which corresponds to the IgG heavy chain. Together, these data expand the repertoire of integrins that bind to Myo10, but lack of binding to the β1 integrin subunit suggests that Myo10-β1 integrin interactions are cell-type dependent.

### Trabecular Meshwork Filopodial Proteins in Response to Mechanical Stretch

**FIGURE 2:**
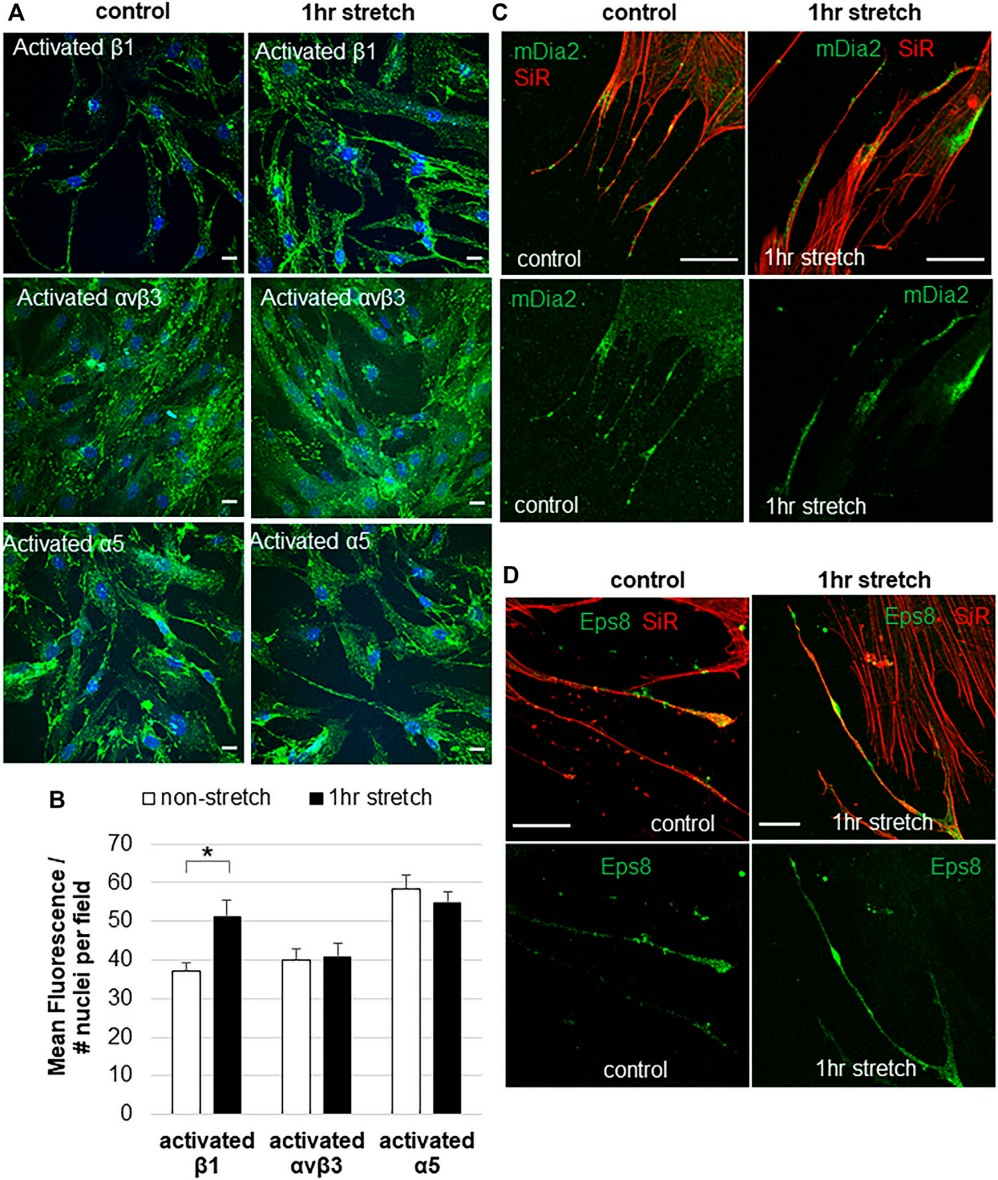
Integrin activation and filopodial proteins in response to 1-hour mechanical stretch. **(A)** Immunostaining of activated β1 integrin (HUTS-21 antibody), activated α5 antibody (SNAKA51), and activated αvβ3 (LIBS2) antibody with and without 1 h mechanical stretch. DAPI stained the nuclei (blue). Scale bar = 20 µm. **(B)** Total fluorescence of activated β1, α5, and αvβ3 integrins was measured in TM cells with and without 1 h mechanical stretch. Relative fluorescence was normalized to the number of nuclei in each field, then data was averaged and standard error of the mean calculated. *N* = 30 images measured. *p = 0.0002 paired students t-test. **(C)** mDia2 was found at the base, along the shaft, and at the tips of TM filopodia, and this distribution was not altered after 1 h mechanical stretch. **(D)** Eps8 tended to accumulate in the filopodia tips. After 1 h stretch, there were several areas of intense Eps8 accumulation in the filopodial shaft as well as the tip.

Mechanical stretch is an *in vitro* method to mimic elevated IOP (Bradley et al., 2001). Integrins may play a central role in detecting changes in mechanical forces caused by elevated IOP (Acott and Kelley, 2008; Gagen et al., 2014; Filla et al., 2017; Acott et al., 2021), but this has not been directly tested. Here, we investigated the effects of a static mechanical stretch on integrin activation. A 1-h mechanical stretch was chosen because live cell imaging shows that mechanosensing filopodia protrude from the TM cell surface in this time frame (Keller et al., 2017; Keller and Kopczynski, 2020). To detect activated integrins, we used HUTS-21, SNAKA51, and LIBS2 conformation-specific monoclonal antibodies, which detect activated forms of β1, α5 and αvβ3 integrins, respectively (Du et al., 1993; Luque et al., 1996; Clark et al., 2005; Byron et al., 2009). There was significantly increased fluorescent signal for activated β1 integrin in TM cells following 1 h mechanical stretch (Figure 2A,B). This increase in activated β1 integrin represented the entire field and was not specifically localized to filopodia. However, there was no difference in the fluorescent signal of activated α5, or activated αvβ3, in stretched vs. non-stretched TM cells. A conformation-specific antibody to activated αvβ5 is not available so we could not assess this subtype.

In addition to integrins, we investigated the distribution of filopodial proteins Eps8 and mDia2 in non-stretched vs. stretched TM cells. In non-stretched TM cells, mDia2 was localized to the base, shaft, and tip of filopodia (Figure 2C), while Eps8 was localized to discrete patches in the shaft and at the tips of filopodia (Figure 2D). mDia2 showed increased areas in the filopodia following mechanical stretch, suggestive of enhanced nascent adhesions.

### Cadherin-11 and Filopodia

**FIGURE 3:**
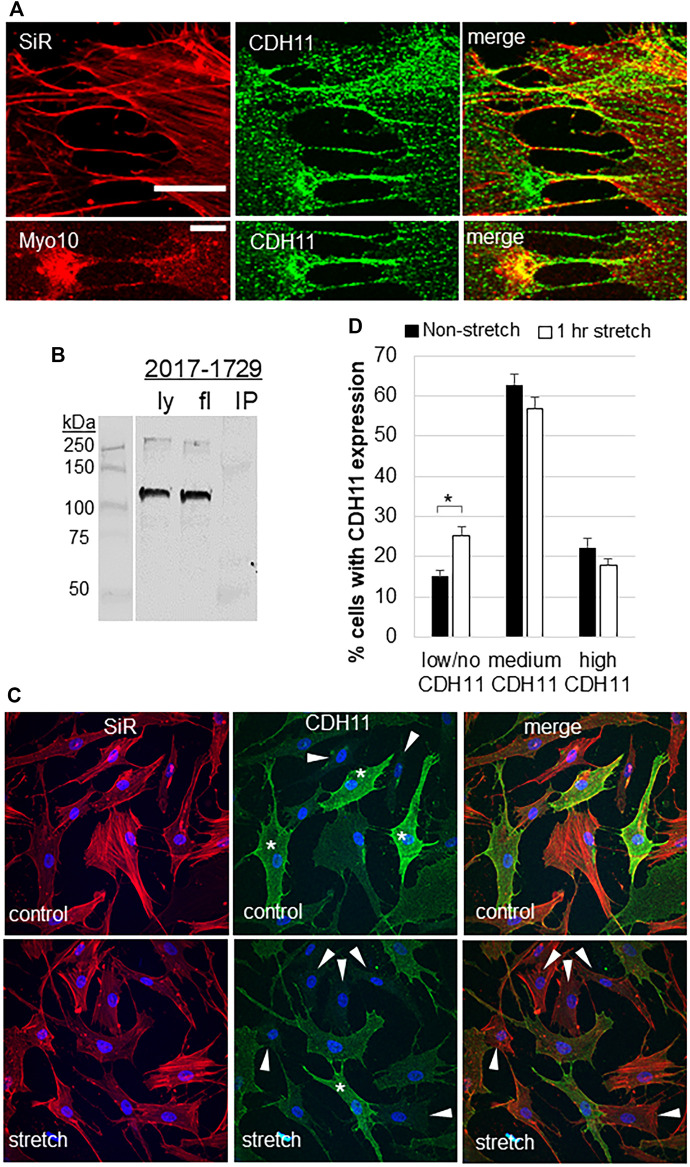
Cadherin-11 in filopodia and in response to mechanical stretch. **(A)** CDH11 was found along the length of filopodia or TNTs connecting adjacent TM cells. CDH11 colocalized with Myo10 at the base of the filopodia, but not along its length. Scale bar = 20 µm. **(B)** CDH11 did not bind to Myo10 in pull-down assays. Ly = lysate; fl = flow-through; IP = immunoprecipitated proteins. **(C)** TM cells were mechanically stretched for 1-h then fixed and stained with CDH11 (green). Actin (red) and DAPI (blue) identified all the TM cells in a field. Cells with high (*), medium, and low/no (arrowheads) CDH11 levels were identified. **(D)** High, medium, and low CDH11 cells were counted and made a percentage of the total number of cells. *p < 0.015 by ANOVA. Data show mean ± s.e.m.

CDH11 is a cell adhesion protein, which is highly expressed in TM cells (Webber et al., 2018). The filopodial protein Myo10 binds to other cadherin family members (Almagro et al., 2010; Lai et al., 2015). Therefore, we investigated CDH11 localization in TM cells. CDH11 localized as punctate dots at the TM cell surface and strongly decorated the base and the entire length of the filopodia (Figure 3A). CDH11 at the filopodial base colocalized with Myo10, but not in the shaft. However, pull-down assays failed to detect an interaction between Myo10 and CDH11 (Figure 3B). We also observed that CDH11 fluorescence was variable, where some TM cells showed high CDH11 levels, others had a medium level of CDH11, while others have little to no CDH11 immunostaining (Figure 3C). We counted the number of high, medium, and low CDH11 cells, with and without 1-h mechanical stretch. Interestingly, the number of low CDH11-expressing cells was significantly higher in stretched than non-stretched cells (Figure 3D) suggesting that mechanical stretch decreased the number of CDH11-positive TM cells.

### Effect of Mechanical Stretch in Normal and Glaucomatous Trabecular Meshwork Cells

**FIGURE 4:**
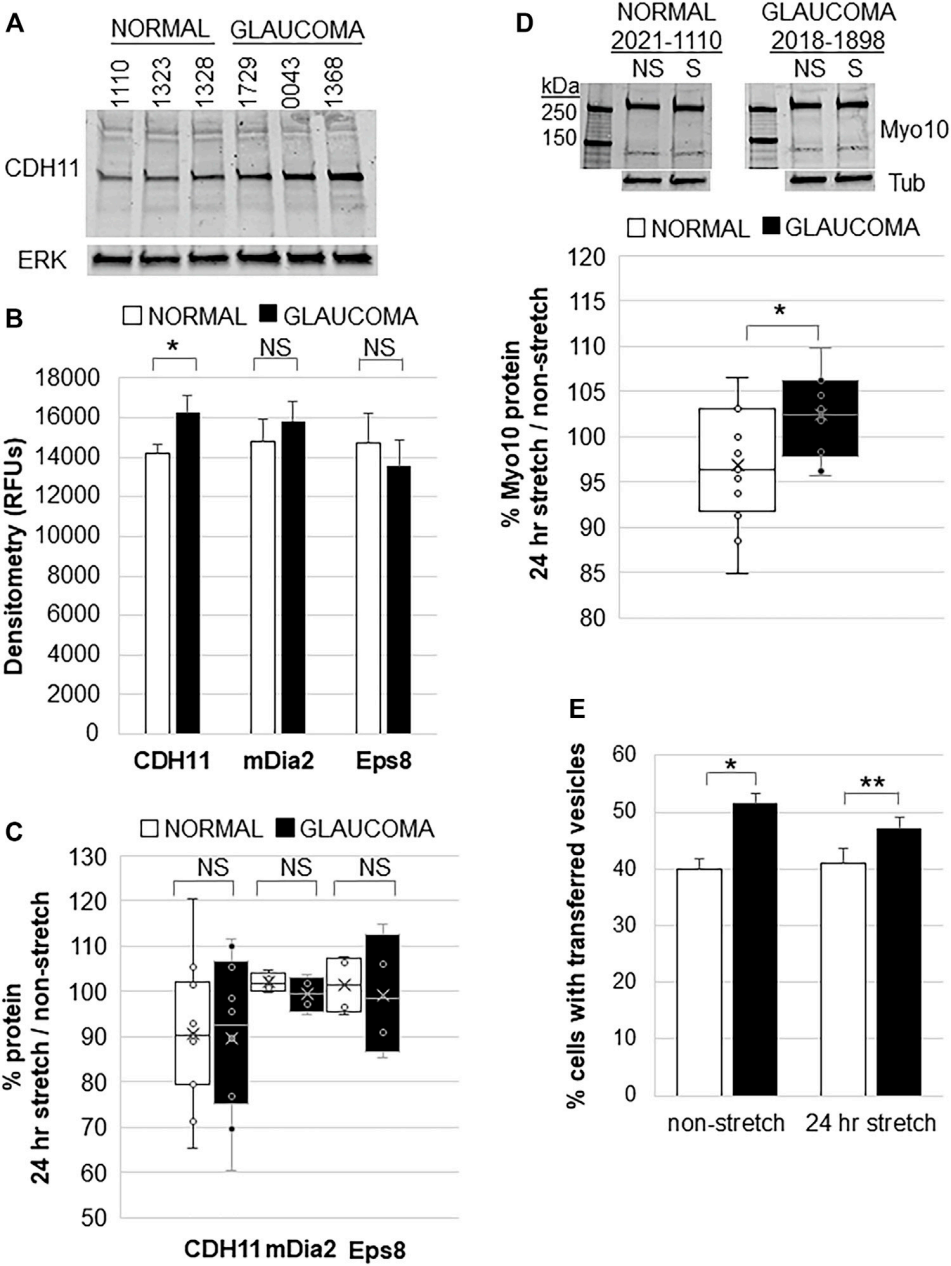
Filopodial proteins in normal and glaucomatous TM cells in response to 24 h mechanical stretch. **(A)** CDH11 protein levels in normal and glaucoma TM cell lysates. ERK1 was used as a loading control. **(B)** Densitometry of CDH11, mDia2, and Eps8 protein levels showed significantly increased levels in glaucoma TM cells. Relative fluorescent units (RFUs) were normalized for ERK loading. Normal: *n* = 17 replicates from 15 NTM cell strains; Glaucoma: *n* = 16 technical replicates from 10 GTM cell strains. *p = 0.038 by ANOVA; NS = not significant. **(C)** Densitometry of CDH11 protein by Western immunoblotting of normal and glaucomatous TM cells after 24 h mechanical stretch. Data show stretch/non-stretched as a percentage. NS = not significant. Normal: *n* = 11 replicates from 10 NTM cell strains; Glaucoma: *n* = 10 technical replicates from 7 GTM cell strains. **(D)** Representative Western immunoblot of Myo10 protein in NTM and GTM cells with and without 24 h mechanical stretch. Tubulin (tub) was used as a loading control. Densitometry showed that GTM cells had a small (∼5%), but significantly increased level of Myo10 protein after 24 h mechanical stretch. *p = 0.023 by ANOVA. Normal: n = 15 replicates from 7 NTM cell strains; Glaucoma: *n* = 10 technical replicates from 5 GTM cell strains. **(E)** TM cells were labeled separately with DiO or DiD dyes, mixed and then mechanically stretched for 24 h. The number of cells containing fluorescent vesicles of the opposite color were counted for normal or glaucomatous cells, with and without stretch. Glaucoma TM cells had significantly increased transfer of vesicles compared to normal TM cells, but mechanical stretch did not cause a significant change. Data show mean ± s.e.m. *p = 0.001; **p = 0.015 by ANOVA.

CDH11 is up-regulated in diseases associated with excess ECM deposition such as invasive breast cancer, scleroderma, and liver fibrosis (Chen et al., 2021). Glaucoma TM tissue has excess ECM so we investigated CDH11 protein levels in normal and glaucomatous TM cells. Western immunoblots and densitometry showed that CDH11 protein levels were significantly increased in GTM cells compared to NTM cells (Figure 4A,B). However, there was no significant difference in mDia2 or Eps8 protein levels between NTM and GTM cells (Figure 4B). We then investigated the effects of 24 h mechanical stretch. There was no difference in CDH11, mDia2, or Eps8 protein levels when comparing stretched vs. non-stretched cells (Figure 4C). Thus, CDH11 is upregulated in glaucoma TM cells, but mechanical stretch does not appear to additionally affect expression levels.

We also investigated whether mechanical stretch affected Myo10 protein levels. Consistent with our previous observations (Sun et al., 2019a), Myo10 levels were not significantly different between non-stretched NTM and GTM cells. However, in response to mechanical stretch, Myo10 protein levels in GTM cells showed a small (∼5%), but significant increase after 24 h compared to NTM cells by Western immunoblot and densitometry (Figure 4D). Over-expression of Myo10 has been shown to induce TNTs (Gousset et al., 2013). Therefore, we hypothesized that increased Myo10 protein levels in mechanically stretched GTM cells may affect cellular communication via TNTs so we performed a fluorescent vesicle transfer assay. In non-stretched cells, GTM cells showed significantly more vesicle transfer compared to NTM cells (Figure 4E), as we found previously (Sun et al., 2019a). Yet, 24 h mechanical stretch did not significantly affect vesicle transfer in NTM cells or GTM cells.

## Discussion

In this study, we show that mechanical stretch, a proxy for elevated IOP, affects the composition and distribution of proteins in TM cell filopodia and vesicle transfer in TNTs. Specifically, we show that TM filopodia contain Myo10, integrins α5β1 and αvβ3, and to some extent αvβ5 integrin, and CDH11, Eps8, and mDia2. Mechanical stretch specifically activated β1 integrin within 1 h, as shown with conformation-specific monoclonal antibodies, suggesting that certain integrins become activated as a rapid response to the stretch and distortion caused by elevated IOP. In addition, mechanical stretch increased the number of CDH11-negative TM cells suggesting that other cell surface adhesion molecules are also affected. In contrast, mechanical stretch had no effect on vesicle transfer via TNTs in either NTM or GTM. Together, these data suggest that filopodial-associated and cell-surface proteins are dynamically altered in response to their biomechanical environment, but these mechanical cues do not significantly impact cellular communication via TNTs. In addition, it provides additional evidence that the function of TNTs and filopodia are different, as previously suggested (Ljubojevic et al., 2021).

Since only heterodimer integrins, not individual subunits, are expressed on the cell surface, and the α5 subunit only pairs with the β1 subunit, localization of the α5 subunit must indicate the presence of the α5β1 integrin heterodimer in filopodia (Hynes, 2002). Localization of α5β1 integrin at the filopodial tip suggests that it is actively engaged with the underlying substrate in a nascent adhesion. α5β1 integrin binding to fibronectin could initiate fibronectin fibrillogenesis or focal adhesion formation in the immediate area, further stabilizing the filopodia (Singh et al., 2010). The discrete areas of α5β1 integrin along the shaft may indicate nascent adhesions (Henning Stumpf et al., 2020). This agrees with the 2-step theory of filopodial elongation, where integrins transiently engage the substrate to stabilize it while additional components are transported toward the tip by Myo10 (He et al., 2017).

Using conformation-specific antibodies (Byron et al., 2009), we showed that mechanical stretch activated β1 integrin, but not α5 or αvβ3 integrins. This suggests that α5β1 integrin may not be involved in detecting mechanical stretch. β1 integrin heterodimerizes with several other α subunits expressed in the TM (Filla et al., 2017). One possibility is α4β1 integrin, which cross-talks with α1β1 and α2β1 integrins (Schwinn et al., 2010; Faralli et al., 2011), and is recognized by the Huts-21 antibody (Njus et al., 2009). Other possibilities are the collagen binding α1β1 or α2β1 integrins (Luque et al., 1996). Further studies are required to reveal the exact identity of integrin heterodimers involved in sensing mechanical stretch-induced alterations to the biomechanical environment.

In this study, we show that Myo10 may play a role in transporting α5β1 integrins to the filopodial tip, as suggested in several cell types (Zhang et al., 2004; He et al., 2017; Miihkinen et al., 2021). The distribution and colocalization of Myo10 with α5 and β1 integrins in filopodia of TM cells supports this idea. However, our pull-down data provide further nuances to Myo10-α5β1 integrin interactions. Myo10 showed positive interaction with the α5 integrin subunit, but not with β1 integrin. This suggests that Myo10 must be interacting with the α5β1 integrin heterodimer via the α5 integrin subunit. An earlier study supports this possibility and found α5 integrin can interact with Myo10 in U2-OS osteosarcoma cells (Miihkinen et al., 2021). Our pull-down assays used the SNAKA51 conformation-specific antibody, thereby showing a positive interaction of Myo10 and activated α5 integrin. Had we used a pan α5-integrin antibody, it is possible that no interaction would have been detected. Nevertheless, our data suggest that different cell types may use either the α5 or the β1 integrin subunit when binding Myo10 (Zhang et al., 2004; Miihkinen et al., 2021).

The pull-down assays also showed positive interactions of Myo10 with αvβ3 and αvβ5 integrins. These data expand the repertoire of integrins that bind to Myo10. αvβ3 integrin is involved in phagocytosis, TGFβ signaling, and is activated by dexamethasone in TM cells (Filla et al., 2011; Faralli et al., 2019; Filla et al., 2021). In addition, αvβ3 integrin activation caused a significant IOP increase in mice (Faralli et al., 2019). Our immunostaining data suggest that Myo10 may traffic αvβ3 integrin to the filopodia tip, like its role for β1 integrin. Also, αvβ3 integrin may be involved in transiently binding to ECM substrates in nascent adhesions in the filopodial shaft and tip (Henning Stumpf et al., 2020). Our prior live imaging studies showed that filopodial tips are cleaved, which allows the filopodia to retract back toward the cell (Keller and Kopczynski, 2020). The cleaved filopodial tips were immunolabeled with several ECM proteins, including αvβ5 integrin. This is consistent with the lack of αvβ5 integrin immunostaining in filopodia shown in this study. It also suggests that Myo10-αvβ5 integrin interactions are important for other TM cell functions.

CDH11 was found associated with filopodia/TNTs connecting TM cells. However, CDH11 immunostaining was not specifically localized to nascent adhesions of the filopodia shaft or tips, but instead, CDH11 was detected along the entire length of the filopodia. Recently, CDH11 was found to localize to focal adhesions, where it binds fibronectin via an interaction with syndecan-4 (Langhe et al., 2016). CDH11 is linked to fibrotic diseases such as liver fibrosis, scleroderma, and pulmonary fibrosis (Chen et al., 2021), and CDH11 is up-regulated by TGFβ2 (Wecker et al., 2013). Therefore, the increased CDH11 protein levels in glaucomatous TM cells is perhaps not surprising. However, we cannot rule out that CDH11 levels may be influenced by prior medications that glaucomatous eyes were subject to. CDH11 forms adhesion complexes, which are dynamically turned over. Here, we show that 1-h of mechanical stretch reduced cell surface CDH11. This could indicate that mechanical stretch induces internalization by TM cells, possibly via clathrin-mediated endocytosis, which is utilized by prostate cancer cells (Satcher et al., 2015). Alternatively, mechanical stretch may induce cleavage of the CDH11 extracellular domain, where the epitope is located. Mechanical stretch and elevated IOP initiates activation of various proteases, including ADAMs and ADAMTSs (Vittitow and Borras, 2004; Vittal et al., 2005). In neural crest cells, ADAMs cleave CDH11 (McCusker et al., 2009), so it is possible that these proteases may cleave CDH11 to reduce the extracellular epitope on the cell surface of mechanically stretched TM cells. Whichever mechanism is utilized, our mechanical stretch data suggest that reduced CDH11 could disrupt cell-cell adhesions and cell-ECM adhesions in TM tissue at elevated IOP.

Myo10 protein levels had small, but significant increases in mechanically stretched GTM cells. During normal filopodia dynamics, Myo10 travels towards the tip of the filopodia during actin assembly and growth, and displays rearward movement as the filopodia is retracted to the cell (Berg and Cheney, 2002). Because actin dynamics are impaired in glaucoma TM cells (Sun et al., 2019a; Keller and Kopczynski, 2020), Myo10 may be expressed longer on glaucomatous filopodia. This may explain increased Myo10 protein levels in mechanically stretch glaucoma cells. However, the lysates were harvested from whole cells, rather than from filopodia specifically. Myo10 also localizes to podosome or invadopodia-like structures (PILS) and PILS are increased by mechanical stretch (Aga et al., 2008; McMichael et al., 2010; Sun et al., 2019b). Thus, the observed increase in Myo10 protein may additionally reflect increased Myo10 at PILS.

In summary, we have shown that Myo10 binds to a wider range of integrins than previously reported. In addition, mechanical stretch specifically and rapidly activates β1 integrin, while reducing CDH11 on TM cell surface. These data provide novel information regarding the cellular structures and transmembrane adhesive proteins that TM cells use to sense changes to the biomechanical environment such as those changes caused by elevated IOP.

## Data Availability

The original contributions presented in the study are included in the article/Supplementary Material, further inquiries can be directed to the corresponding author.
