# Supplementary figures and images for: The Effects of Mechanical Stretch on Integrins and Filopodial-Associated Proteins in Normal and Glaucomatous Trabecular Meshwork Cells
Source: Front Cell Dev Biol. 2022 Apr 29;10:886706. doi: 10.3389/fcell.2022.886706 (PMC9100841; doi:10.3389/fcell.2022.886706)

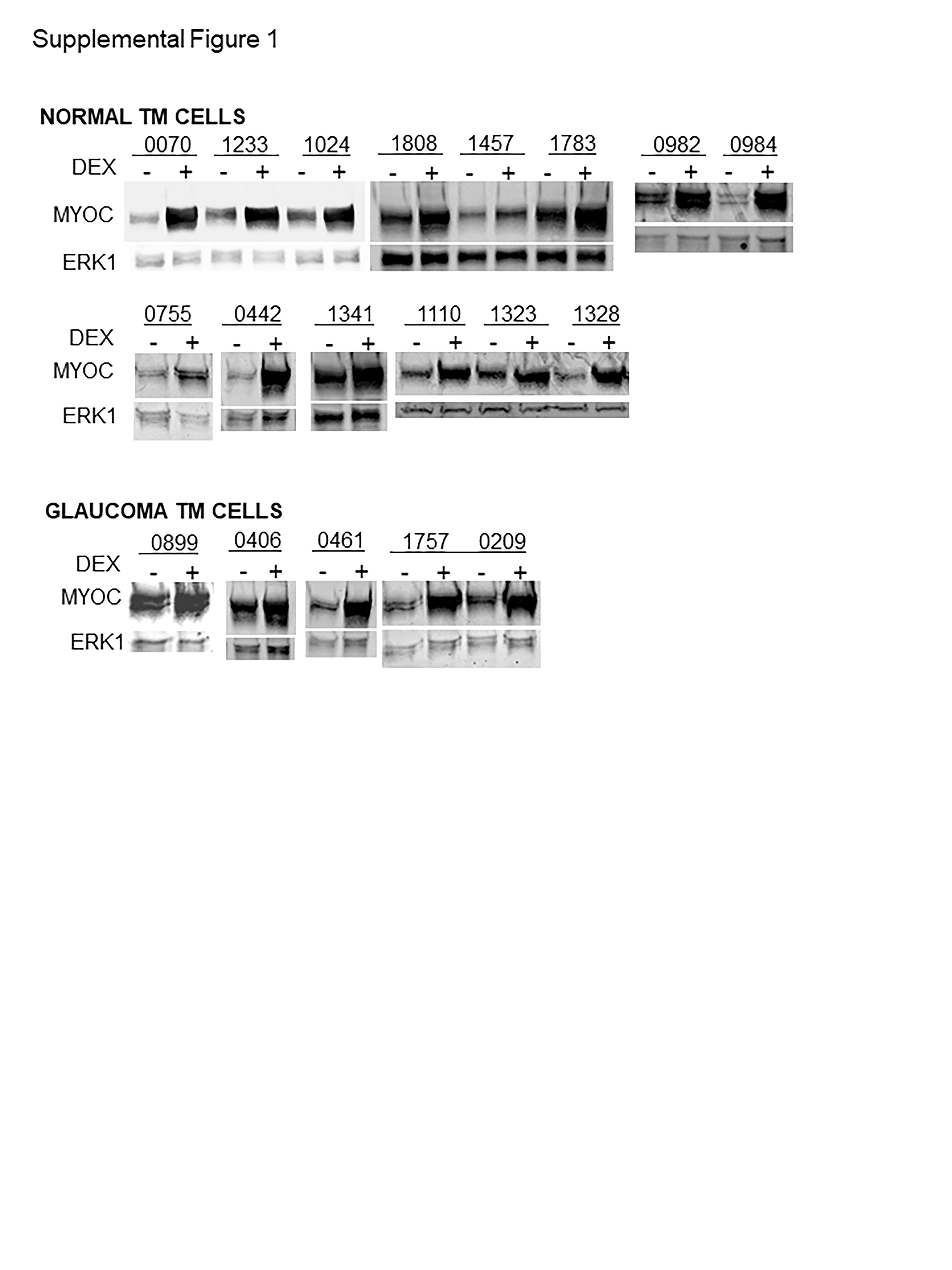

Supplement: Supplementary file 1 [file Image1.TIF]
