# Supplementary material for: The Effects of Mechanical Stretch on Integrins and Filopodial-Associated Proteins in Normal and Glaucomatous Trabecular Meshwork Cells
Source: Front Cell Dev Biol. 2022 Apr 29;10:886706. doi: 10.3389/fcell.2022.886706 (PMC9100841; doi:10.3389/fcell.2022.886706)
Supplement: Supplementary file 2 [file DataSheet1.PDF]

Supplementary Table 1: Demographics of the donor eyes.

**NORMAL**				
**Cell Strain**	**Age**	**Sex**	**Cause of Death**	
2018-0070	54	M	cardiac arrest	
2018-1233	53	M	lung CA w/ metastases	
2019-1024	18	M	polytrauma with traumatic brain injury	
2011-1808	19	M	Multiple trauma	
2012-1457	47	M	Ventricular fibrillation arrest	
2018-1783	38	M	respiratory failure	
2020-0982	44	M	Interstitial lung disease	
2020-0984	69	M	Advanced Parkinson's and dementia	
2021-0755	21	F	Respiratory failure	
2021-1110	77	F	Lung cancer w/ metastases	
2021-1323	57	M	cardiac arrest	
2021-1328	75	M	cardiac arrest	
2014-0442	8mo	M	Respiratory failure	
2018-1341	55	M	myocardial infarction	
**GLAUCOMA**				
**Cell Strain**	**Age**	**Sex**	**Cause of Death**	**Glaucoma Status**
2019-1757	92	F	cardiogenic shock	POAG OU (latanoprost gtt OU)
2020-0209	74	F	acute diastolic heart failure	primary open-angle glaucoma OU
2020-0899	94	F	acute diastolic heart failure	primary open-angle glaucoma OU
2019-0406	81	F	septic shock	open angle glaucoma OD
2019-0461	96	F	non ST elevation myocardial infarction	Glaucoma OU (brimonidine & latanoprost gtt OU)

Supplementary Table 2: Commercial sources of antibodies used.

**Protein Name**	**Company**	**Catalog #**
Myosin-X rabbit polyclonal	Invitrogen	PA5-55019
Cadherin-11 mouse monoclonal	Invitrogen	MA1-06306
CD44 rat monoclonal	Stem Cell Tech.	60068
mDia2 (Diaph3) rabbit polyclonal	MilliporeSigma	HPA032151
Eps8 mouse monoclonal	Abnova	H00064787-M01
Integrin-beta 1 rabbit polyclonal	MilliporeSigma	AB1952
Integrin-beta 1 mouse monoclonal, clone P5D2	MilliporeSigma	MAB1959
Activated integrin-beta 1 mouse monoclonal, clone HUTS-21	BD Biosciences	556048
Integrin-alpha 5 rabbit polyclonal	Cell Signaling Tech.	4705T
Activated alpha 5 mouse monoclonal, clone SNAKA51	MilliporeSigma	MABT201
Integrin alpha V beta 3 mouse monoclonal	Abcam	ab78289
Activated integrin alpha V beta 3 mouse monoclonal, clone LIBS2	MilliporeSigma	MABT27
Integrin alpha V beta 5 mouse monoclonal	Abcam	ab177004
Total ERK1 rabbit polyclonal	MilliporeSigma	M5670
Tubulin mouse monoclonal	MilliporeSigma	T6199
Myocilin mouse monoclonal	Santa Cruz<br>R&D Systems	Sc137233<br>MAB3446
